# The influence mechanisms of inclusive leadership on job satisfaction: Evidence from young university employees in China

**DOI:** 10.1371/journal.pone.0287678

**Published:** 2023-06-23

**Authors:** Huiqian Li, Cheng Zhou

**Affiliations:** 1 Personnel Department, Yulin Normal University, Yulin, 537000, Guangxi, P. R. China; 2 Business School, Nanjing University of Science and Technology ZiJin College, Nanjing, 210023, Jiangsu, P. R. China; 3 School of Public Administration, Nanjing Normal University, Nanjing, 210023, Jiangsu, P. R. China; Jiangsu University, CHINA

## Abstract

**Background:**

Leadership style and job satisfaction are currently hot issues in the field of management psychology research, especially with regard to young employees.

**Objective:**

This study attempted to explain the mechanism of improving employees’ job satisfaction by combining the key factors of work-family balance and psychological capital.

**Methods:**

We adopted the literature method, questionnaire survey method, and statistics method to conduct the research. And we conducted structural equation modeling (SEM) analysis of 540 young university employees in China using the random sampling method for sampling.

**Results:**

Based on the structural equation modeling (SEM) analysis of 540 young university employees in China, the results show that inclusive leadership has a positive impact on improving employees’ job satisfaction and that work-family balance is beneficial to serving leaders in improving employees’ job satisfaction. Simultaneously, psychological capital positively moderates the indirect effect of inclusive leadership on improving job satisfaction.

**Conclusion:**

The final model revealed an important path from inclusive leadership to job satisfaction through work-family balance. These findings not only extend and enrich the relevant research on the relationship between inclusive leadership and job satisfaction but also shed some light on university management practice.

## Introduction

Teams are composed of leaders and employees, and leadership style has a significant impact on team members’ job satisfaction. University employees, as the organizational team for colleges and universities, shoulder the important mission of cultivating higher talents. The job satisfaction of university employees has an important impact on their professional enthusiasm and efficiency. University employees have a diverse knowledge base, so it is particularly important to improve their job satisfaction. The leadership style must shift from one of control, hierarchy, and rule-following to one of inclusive leadership [1]. Tolerance is a historical experience in the development of Chinese culture and an important idea in the UN Millennium Development Goals. Tolerance is the driving force for social change in China and a symbol of contemporary civilization. Inclusive leadership is a leadership style that emphasizes the interdependence of leaders and employees. With this leadership style, leaders pay attention to the needs and interests of employees and cooperate with them to better develop their potential and energy, thereby producing a certain degree of job satisfaction. Therefore, this study took the university worker team as the research object, combined with the work characteristics of the university worker group, took the two dimensions of work-family balance and psychological capital as mediating variables, explored the influence of leadership style on job satisfaction of university employees, and put forward targeted improvement suggestions in order to promote the team of university employees to be more harmonious and close and improve their work efficiency and job satisfaction.

Research on inclusive leadership stems from inclusive management. The inclusive leadership style is a brand-new leadership behavior construct proposed by Michael in the field of organizational behavior, based on Maslow’s hierarchy of needs theory from the late 1970s [2]. Rucker (1985) explained it as referring to giving individualized care to subordinates, and subordinates’ leadership style and leadership behavior have an important impact on employee job satisfaction [3]. According to Anand and Yonghwa (2008), inclusive management in the team should include inclusive culture, inclusive leadership, inclusive professional practice, and so on [4]. Guo and Hu (2022) summarized the views of current scholars at home and abroad on this basis and defined inclusive management. Individual differences encourage employees to express themselves and contribute their unique value, which improves overall organizational performance through the integration of individual human resources [5]. Malak et al. (2019) pointed out that inclusive leadership should mean that leaders invite and appreciate the input of employees and affirm their contributions so that employees feel valued and respected. Behavior can reduce the differences caused by team heterogeneity, effectively promote team members’ cooperation, and improve workflow [6]. In 2022, scholars such as Bakri et al. further defined inclusive leadership based on this theory, arguing that inclusive leadership is a specific form of relational leadership, which refers to openness and accessibility in interactions with subordinates [7]. Another study investigated sexuality and the availability of leadership behavioral characteristics. In 2022, Lin and other scholars, in their research on the relationship between inclusive leadership style and employees’ innovative behavior, proposed that inclusive leadership style refers to the attitude and behavior of leaders who are open, friendly, tolerant, and supportive at work [8].

With the extensive research on the effects of leadership style on employee job satisfaction, some scholars have gradually begun to pay attention to the exploration and research of worker groups. Gao (2021) proposed that an inclusive leadership style can make managers of worker groups realize their responsibilities, thereby stimulating their pursuit of work, social status, and self-worth and improving the overall education of worker organizations, resulting in leadership behaviors that exceed the originally expected results. They pointed out that inclusive leadership styles should be reflected in four aspects in the worker group: charisma, individualized care, intellectual stimulation, and idealized influence [9]. Sfakianaki et al. (2018) showed that leadership style has a significant positive correlation with the work attitude, work behavior, and organizational performance of the worker group, which can be generated by encouraging employees to switch their interests to organizational interests. This study has a far-reaching impact [10]. Xia and Zhang (2022) made further research on this and proposed that the role of inclusive leadership style in the working group in promoting job satisfaction is mainly reflected in the vision, motivation, and moral example in the leadership style. Significant impact, leadership charisma, and individualized care have a significant impact on worker satisfaction [11]. Jin et al. (2020) showed that an inclusive leadership style significantly positively affects the work engagement of employees, and the principal’s leadership style can significantly predict employee job satisfaction [12]. Cao’s (2021) research on the influence of domestic primary and secondary school leadership style on employees’ job satisfaction found that leadership style and employees’ work engagement, employees’ job satisfaction, employees’ task-based psychological capital, and other variables were positively correlated [13].

The influence of an inclusive leadership style on the job satisfaction of employees is also influenced by the mediating factors of psychological capital and work-family balance. In 2018, research by Zhou et al. (2018) showed that psychological capital is positively correlated with the job satisfaction of employees and can predict the variation of various factors of work engagement [14]. Kuan et al. (2017) studied the relationship between psychological capital and the job satisfaction of middle school workers and found that both resilience and optimism in psychological capital had a significant positive impact on job satisfaction [15]. Mao and Tan (2015) surveyed primary and secondary school workers and showed that both the psychological capital and the family field of workers significantly promoted their job satisfaction [16].

In recent years, more and more researchers have begun to focus their research on the relationship between leadership style and workers’ work attitude, work behavior, and job satisfaction and have proposed the influence of mediating variables such as psychological capital and work-family balance from the perspectives of social exchange, social learning, and psychological cognition. However, research on the relationship between inclusive leadership and job satisfaction for the group of university workers is currently lacking, particularly research on how the relationship is affected by the mediating factors of work-family balance and psychological capital. This research is significant because educators have a professional, in-depth, and expansive organizational level where the directional requirements of leaders will be stricter and the leadership style will affect the workers. Therefore, the impact will likely be more far-reaching. It is, therefore, important to explore and study the relationship between job satisfaction and the leadership style of university workers in order to enrich the leadership strategies of university management teams and improve the level of education delivered.

This is because inclusive leadership is an innovative and up-to-date leadership style that provides a fresh approach to employee diversity issues in today’s universities. Based on the previous research results of scholars, the research took university employees as the object and used empirical methods to explore the influence of factors such as work-family balance and psychological capital on leadership style and university employees’ job satisfaction, which is the research objective. The research questions of this study were: (1) Will inclusive leadership of university employees have a direct impact on their work attitude? (2) Will the mediating factors of work-family balance and psychological capital affect the influence of inclusive leadership on the job satisfaction of university employees?

Job satisfaction will have a significant impact on the job engagement and work efficiency of university employees, and leadership style is closely related to the job satisfaction of university employees. Human resources management in universities should not only strengthen the management of university teachers but also focus on cultivating team leaders who reflect an inclusive leadership style that keeps pace with the times. In the current economic environment, the diversification of the team of university employees requires changes in the management of leaders, and the way of leading should be more flexible. The contributions of the study are to let university leaders realize the important role of inclusive leadership in the university team and the mediating role of work-family balance and psychological capital. Some suggestions will help them innovate their leadership styles, deepen the connection between the university employees, improve their professional identity and job satisfaction, and help achieve the common growth of university organizations and individuals.

## Literature review and hypotheses development

### 1. Influence of inclusive leadership style on job satisfaction

Reed and Lewin (1951) used field theory as a method of analyzing and dealing with problems. He believes that the psychological field where human behavior occurs is the living space. People and the environment are seen as a dynamic whole. This theory serves as the premise of this study and guides it [17]. With the introduction of the concept of inclusive leadership style, scholars have gradually begun to focus on researching the impact of inclusive leadership style on employee job satisfaction. Sridevi (2019) concluded that an inclusive leadership style can improve the performance of individual employees and their enthusiasm for work through a qualitative analysis of enterprises and believed that an inclusive leadership style is reflected in the performance and appreciation of employees [18]. Through a questionnaire survey, Crews et al. (2019) conducted research on the influence of leadership style in the hospital on the job satisfaction of the medical and nursing teams. The study found that an inclusive leadership style can help improve the work enthusiasm of the medical and nursing teams. It can also improve the prognosis and recovery degree of patients, and the questionnaire used by this scholar has been widely recognized and applied in China. This questionnaire includes three dimensions, namely openness, usability, and accessibility [19]. In 2021, Yang conducted interviews and research on managers with more than 15 years of experience in the enterprise and finally extracted that the influence of an inclusive leadership style on employees’ job satisfaction is mainly reflected in “support” “recognition” “communication” “action” “fairness” “respect” and other aspects [20]. From the perspective of new-generation employees, Zhang (2021) studied the influence of an inclusive leadership style on team job satisfaction in listed companies. The scholar proposes that inclusive leadership styles should be divided into balanced delegation, walking management, and progressive. They found three modes of sexual innovation and found that an improvement in leadership style has a positive effect on team job satisfaction and can improve team cohesion [21].

At present, no scholars at home or abroad have studied the impact of an inclusive leadership style on job satisfaction among university employees. The existing research mainly focuses on studying transformational leadership styles. For example, Tian et al. (2020) investigate the impact of transformational leadership on employee retention in small- and medium-sized enterprises (SMEs). The findings of the study reveal a positive and significant relationship between transformational leadership and organizational citizenship behavior [22]. Sikandar et al. (2022) address how SMEs’ potential effectiveness is derived from their employees when leadership plays its role efficiently and promotes voluntary work among employees of Pakistani SMEs. The findings of the study find that organizational citizenship behavior and transformational leadership have an effect on engaging employees in voluntary work that ultimately improves performance in SMEs [23]. However, Li et al. (2020) conducted research on the managers of scientific research teams in domestic universities. They found that the management style will affect the scientific research level of the team of university employees, and it is believed that the inclusive leadership style should be reflected in the cultivation and recognition of team members, enabling team members to give full play to their advantageous roles and achieving mutual benefit and win-win results [24]. In addition, Reed and Lewin’s field theory (1951) proposed that people associate their identity and self-esteem with the group or organization they belong to [17]. In the workplace, the identity of employees is usually associated with their team or department. Therefore, when adopting an inclusive leadership style in an organization, leaders will respect employees’ different backgrounds and cultures and create a supportive and encouraging working environment. Employees will feel more identified with and engaged in the organization, which can enhance their job satisfaction. Therefore, we hypothesize:

H1
Inclusive leadership style impacts job satisfaction.

### 2.Mediating effect of work-family balance between inclusive leadership style and job satisfaction

Reed & Lewin (1951) suggested that the psychological field theory mainly includes three meanings: first, people and the environment are the basic elements that constitute the living space, and only when the environment is combined with people’s psychological goals and the role of the environment is brought into play can the living space be formed; Second, the living space has the function of power, which is mainly manifested in two kinds of attraction and repulsion, and the function of this kind of power can drive individuals to overcome the repulsion and move towards the psychological goal along the direction of attraction. Third, the dynamic function of the living space is to gradually play a role, through which the individual gradually overcomes obstacles and achieves the ultimate goal [17]. With rapid economic development and the social trend of innovation, current employees are under huge work pressure. The busy and tense work results in most employees having to face the problem of balancing work and family, which affects their performance and career. Various aspects, such as well-being, have had an important impact. Nurani et al. (2022) focused on the status quo and influencing factors of work-family balance among entrepreneurs. The results showed that an inclusive leadership style has a significant positive impact on entrepreneurs’ work-family balance and psychological empowerment. Playing a mediating role between the two, the scholar proposed that work-family balance is a common problem encountered in the process of entrepreneurship and that simply reducing work time or workload is not a good strategy to achieve work-family balance [25]. Li (2015) conducted a study on the influence of employees’ work-family balance on their creativity. The results of the study showed that a good work-family balance will have a positive impact on employees’ creativity at work, but this will be affected by the regulation of the leadership style. The scholar pointed out that there is a positive spillover effect and a multiplicative effect between work and life, and employees should pursue a higher level of work-family balance. Therefore, it is important to increase the psychological empowerment of employees through inclusive leadership styles so that employee autonomy leads to increased job satisfaction, which in turn promotes an improvement in quality of life, and this cycle continues [26]. Ma (2015) conducted a study on the leadership styles of female managers in schools, and the results showed that female managers were more inclined to use an inclusive leadership style, and most of them were able to balance the relationship between home and work [27]. Du and Yu (2016) researched female managers in enterprises and found that, compared with male managers, the leadership style of female managers has a more significant impact on the employees’ work-family balance [28].

Work and family are two important areas of people’s lives, and the relationship between them will have a direct impact on the job satisfaction of employees in modern organizations. Baqutayan and Ariffin (2017) found that job satisfaction has a direct impact on employees’ willingness to leave, and this will be affected by factors such as family resources, work enthusiasm, job performance, and organizational management behavior. The scholars pointed out that a good work-family balance will have a significant impact on employee job satisfaction [29]. Fiebig and Christopher (2018) showed that the work-family balance of enterprise employees has a significant positive correlation with job satisfaction and that employees’ job satisfaction will have a positive incentive effect on their active work, thereby improving enterprise performance and promoting the development of the market economy [30]. At the same time, scholars such as Li et al. (2021) have developed empirical research on the factors that affect employee job satisfaction in enterprises. The family field will play a role in promoting the work field, and promoting employees’ work-family balance will help improve employees’ job satisfaction. They also propose that leadership style will have a positive impact on the workplace. According to the important social exchange theory, increasing job satisfaction will reduce employees’ turnover intentions, which will make employees work harder, resulting in a two-way positive impact on the family field [31]. Imonikhe and Lukic (2022) found that the effective cycle of job satisfaction and employees’ turnover intention mainly depends on the balance between employees’ performance and work-family relationships, and the employees’ family situation has a direct impact on performance levels. In terms of job performance, employees’ work attitudes and work behaviors have a two-way impact, i.e., their attitude will affect their work behavior, which in turn affects their performance level [32]. Employees’ work-family conflict, according to Xing and Li (2020), will have a negative impact on their job satisfaction, while an improvement in organizational team leadership style and teamwork within the company will have a positive impact on their work-family relationship [33]. According to Reed and Lewin’s field theory (1951), events or experiences in the living and working environments may interact and form a cumulative effect [17]. Therefore, when employees feel that the balance between work and family is well maintained, they will feel more relaxed and joyful, thus having more energy and motivation to face the challenges in their work. This can improve employee job satisfaction. Consequently, we hypothesize:

H2
Work-family balance has a mediating effect between inclusive leadership style and job satisfaction.

### 3. Moderating effect of psychological capital between inclusive leadership style and job satisfaction

Based on human capital and social capital, psychological capital, as new capital, can reflect the competitiveness of individuals. This refers to an individual’s positive mental abilities that could improve job performance. Foreign studies have shown that the overall structure or individual factors of psychological capital can improve individual job satisfaction, organizational commitment, political success, sales performance, leadership effectiveness, strategic decision-making effectiveness, and performance, thereby reducing employee work stress and absenteeism. Psychological capital enhances the optimism, retention tendency, and job satisfaction of subordinates; reduces the absenteeism rate of subordinates; promotes the effectiveness of organizational reform; increases the number of enterprises founded; and improves the resilience, profitability, and company performance of the organization [34]. The theory of psychological capital has received positive responses from scholars in China. Using foreign scales, scholars such as Yang Weiguo found that psychological capital has a positive impact on employees’ organizational commitment, organizational citizenship behavior, and job performance. Taking cultural differences into account, scholars such as Ke et al. (2022) have developed a local scale that includes transactional psychological capital (confidence and courage, optimism and hope, perseverance and tenacity) and interpersonal psychological capital (tolerance and forgiveness, respect and comity, humility, sincerity, stability, and gratitude). The scale has two dimensions: one is similar to Western psychological capital, and the other has a unique local cultural flavor [35]. The empirical results support the study’s view that the local scale has better reliability and validity than the Western scale of Luthans et al. and has a stronger explanatory power for job performance, especially peripheral performance. According to this scale, many domestic scholars have proven the positive effect of psychological capital on employees and university students. Even in an integrated framework, Ke Jianglin et al. proved that the impact of psychological capital on job performance exceeded the sum of human and social capital and was more effective in the context of uncertain tasks. Reed and Lewin’s field theory (1951) also provided a theoretical basis, stating that people are more likely to feel satisfied when meeting needs in their environment [17]. In the workplace, employees’ psychological capital and needs include receiving support from the organization and leaders, gaining respect and recognition, and unleashing their abilities and potential. Inclusive leaders listen to employees’ opinions and suggestions and provide support and guidance, which can help employees meet these needs and improve their job satisfaction. Thus, we hypothesize:

H3
Psychological capital has a moderating effect between inclusive leadership style and job satisfaction.

## Methods

### (1) Research objects

The data for this study came from universities in Guangxi Province, China. A total of 580 young university employees born between 1985 and 1995 were randomly selected to participate in a questionnaire survey. Personnel engaged in scientific research and teaching in universities are included in the research object, as are teachers who are primarily teachers but also serve administrative functions, and non-teaching personnel specializing in administrative and management work, such as teaching assistants and office management positions. The questionnaires used were the Hollander (2009) scale, the Luthans (2007) scale, the work-family balance scale developed by Crzywacz and Marks (2000), and the Minnesota Short Form (1967). Survey respondents answered questions about inclusive leadership, psychological capital, job balance, and job satisfaction. The scales used were all translated following standard literal and back-translation procedures. All the participants in the survey completed the questionnaire on the spot. After removing the invalid questionnaires, 580 questionnaires were approved, and the final response rate of the questionnaires was 74%.

### (2) Methods

#### 1. Investigation type

The study was conducted by questionnaire. Before the investigation, we read and sorted the existing domestic and foreign literature, investigated the relationship between inclusive leadership, psychological capital, job balance, and job satisfaction, and put forward relevant hypotheses.

#### 2. Investigation design

We selected a wide range of mature scales, selected a small sample for pre-investigation, and clarified the rationality of the questionnaire. Then, the formal questionnaires were distributed, and the formal samples required for empirical analysis were obtained by strictly controlling the filling process and carefully screening the valid questionnaires.

#### 3. Investigation scope

The survey covered college workers born from 1985 to 1995 in Guangxi, China. The survey was conducted from January to April 2022. The study used SPSS 25.0 and Amos 24 to process and analyze the formal samples, and the hypotheses were verified by SEM analysis.

### (3) Ethical statement

In this study, the rights and interests of the respondents are fully protected, conforms to the requirements of the Ethics committee and the provisions of the Declaration of Helsinki (as revised in Brazil 2013). All participants gave verbal consent to take the survey (Opinions about job satisfaction, etc. No personal information is involved.), and all data were processed anonymously. Agree to the research protocol.

## Results

### (1) Descriptive results

#### 1. Population frequency analysis

Among all the people who participated in the survey, 50.86% were male and 49.14% were female; 68.62% had a master’s degree or above, and these statistical data conformed to the characteristics of the personnel system of university employees (Table 1).

**Table 1:** Frequency analysis results.

Title	Category	Frequency	Percent (%)	Cumulative percentage (%)
Gender	Male	295	50.86	50.86
	Female	285	49.14	100.00
Post	Administration	202	34.83	34.83
	Teacher	206	35.52	70.34
	Other	172	29.66	100.00
Marital status	Married	280	48.28	48.28
	Unmarried	300	51.72	100.00
Highest education	Undergraduate	183	31.55	31.55
	Master	215	37.07	68.62
	Above	182	31.38	100.00
Working years	3 years or less	200	34.48	34.48
	4 years	150	25.86	60.34
	5 years	140	24.14	84.48
	5+ years	90	15.52	100.00
Total		580	100.0	100.0

#### 2. Reliability analysis

The three dimensions of inclusive leadership had a reliability of 0.887, 0.897, and 0.906, proving that the reliability was excellent. The four dimensions of the work-family balance had a reliability of 0.881, 0849, 0871, and 0.848, proving that the reliability was notable. The two dimensions of job satisfaction had a reliability of 0.947 and 0.937, proving that the reliability was significant. The four dimensions of psychological capital had a reliability of 0.913, 0.914, 0.916, and 0.919, proving that the reliability was excellent (Table 2).

**Table 2:** Reliability analysis.

Cronbach’s Alpha	Inclusive Leadership	Recognition and support	0.887
		Fairness in communication	0.897
		Self-disrespect	0.906
	Work-Family Balance	Work interferes with family	0.881
		Home promotion work	0.849
		Family interference with work	0.871
		Work promotes family	0.848
	Job Satisfaction	Internal satisfaction	0.947
		External satisfaction	0.937
	Psychological Capital	Self-efficacy	0.913
		Hopefulness	0.914
		Resilience	0.916
		Optimism	0.919

#### 3. Validity test

It can be seen from Table 3 that the KMO results were greater than 0.6, which met the prerequisite requirements of factor analysis, meaning that the data could be used for factor analysis research. In addition, the data passed the Bartlett sphericity test (p < 0.05), indicating that the research data were suitable for factor analysis (Table 3).

**Table 3:** KMO and Bartlett’s test.

		Inclusive leadership	Work-family balance	Job satisfaction	Psychological capital
KMO		0.946	0.913	0.964	0.955
Bartlett’s test	Approx. Chi-Square	5908.217	4594.523	8444.660	10323.738
	df	120	91	190	276
	p	0.000	0.000	0.000	0.000

#### 4. Confirmatory factor analysis

In the confirmatory factor analysis, according to the criteria, the value of χ2/df was less than 5; GFI, CFI, and NFI were greater than 0.9; and the RMSEA was less than 0.1; the results, therefore, indicate that the confirmatory factor analysis results were good (Table 4).

**Table 4:** Model fit metrics.

Common indicators	χ2	df	p	χ2∕df	GFI	RMSEA	RMR	CFI	NFI	NNFI	Default model
Inclusive leadership	313.192	101	0.000	3.101	0.927	0.060	0.035	0.964	0.948	0.957	χ2(120) = 5982.135, p = 1.000
Work-family balance	132.615	71	0.000	1.868	0.967	0.039	0.028	0.986	0.971	0.983	χ2(91) = 4646.597, p = 1.000
Job satisfaction	452.083	169	0.000	2.675	0.920	0.054	0.036	0.966	0.947	0.962	χ2(190) = 8570.259, p = 1.000
Psychological capital	886.878	246	0.000	3.605	0.857	0.067	0.046	0.937	0.916	0.930	χ2(276) = 10501.785, p = 1.000
Overall	61.461	59	0.388	1.042	0.984	0.008	0.017	0.999	0.981	0.999	χ2(78) = 3214.280, p = 1.000.

### (2) Inferential results

#### 1. Correlation analysis

Correlation analysis was used to study the correlation between inclusive leadership and psychological capital, work-family balance, and job satisfaction, and the Pearson correlation coefficient was used to indicate the strength of the correlation. The specific analysis showed that inclusive leadership and psychological capital, work-family balance, and job satisfaction all had significant differences, and the correlation coefficient values were 0.447, 0.577, and 0.491, respectively (Table 5), with all the correlation coefficient values being greater than 0. This means that there was a positive relationship between inclusive leadership and psychological capital, work-family balance, and job satisfaction.

**Table 5:** Correlation analysis.

	Mean	S.D.	Inclusive Leadership	Psychological capital	Work-family balance	Job satisfaction
Inclusive leadership	3.377	0.784	1			
Psychological capital	3.228	0.795	0.447**	1		
Work-family balance	3.323	0.757	0.577**	0.320**	1	
Job satisfaction	3.281	0.802	0.491**	0.408**	0.529**	1

Note: * p < 0.05; ** p < 0.01.

#### 2. Linear regression analysis

When we ran a linear regression with inclusive leadership as the independent variable and job satisfaction as the dependent variable, the mediation effect involved two model equations (Table 6), which are as follows:

Job satisfaction = 2.331–0.298*Inclusive leadership-0.347*Psychological capital+0.163*Inclusive leadership*Psychological capital+0.376*Work-family balance ①

Work-family balance = 1.443 +0.557*Inclusive leadership ②

**Table 6:** Linear regression analysis results.

	Job Satisfaction				Work-family balance			
	B	SE	t	p	B	SE	t	p
Constant	2.331	0.421	5.537	0.000**	1.443	0.114	12.688	0.000**
Inclusive leadership	-0.298	0.128	-2.321	0.021*	0.557	0.033	16.974	0.000**
Psychological capital	-0.347	0.140	-2.485	0.013*				
Inclusive leadership* Psychological capital	0.163	0.039	4.140	0.000**				
Work-family balance	0.376	0.043	8.827	0.000**				
N	580				580			
R^2^	0.385				0.333			
Adjust R^2^	0.379				0.330			
F	F (4,575) = 89.832, p = 0.000				F (1,578) = 288.123, p = 0.000			

Note: * p < 0.05; ** p < 0.01.

It can be seen that inclusive leadership with psychological capital and work-family balance had a significant impact on job satisfaction in Eq ①. The interaction item between inclusive leadership and psychological capital showed significance (p = 0.000<0.05), which means that when inclusive leadership affects job satisfaction, the moderating variable (psychological capital) is significantly different at different levels. Then, combined with the results of Eq ②, it can be seen that the indirect effect of the model was significant, meaning that it had a mediating effect.

**Fig 1:**
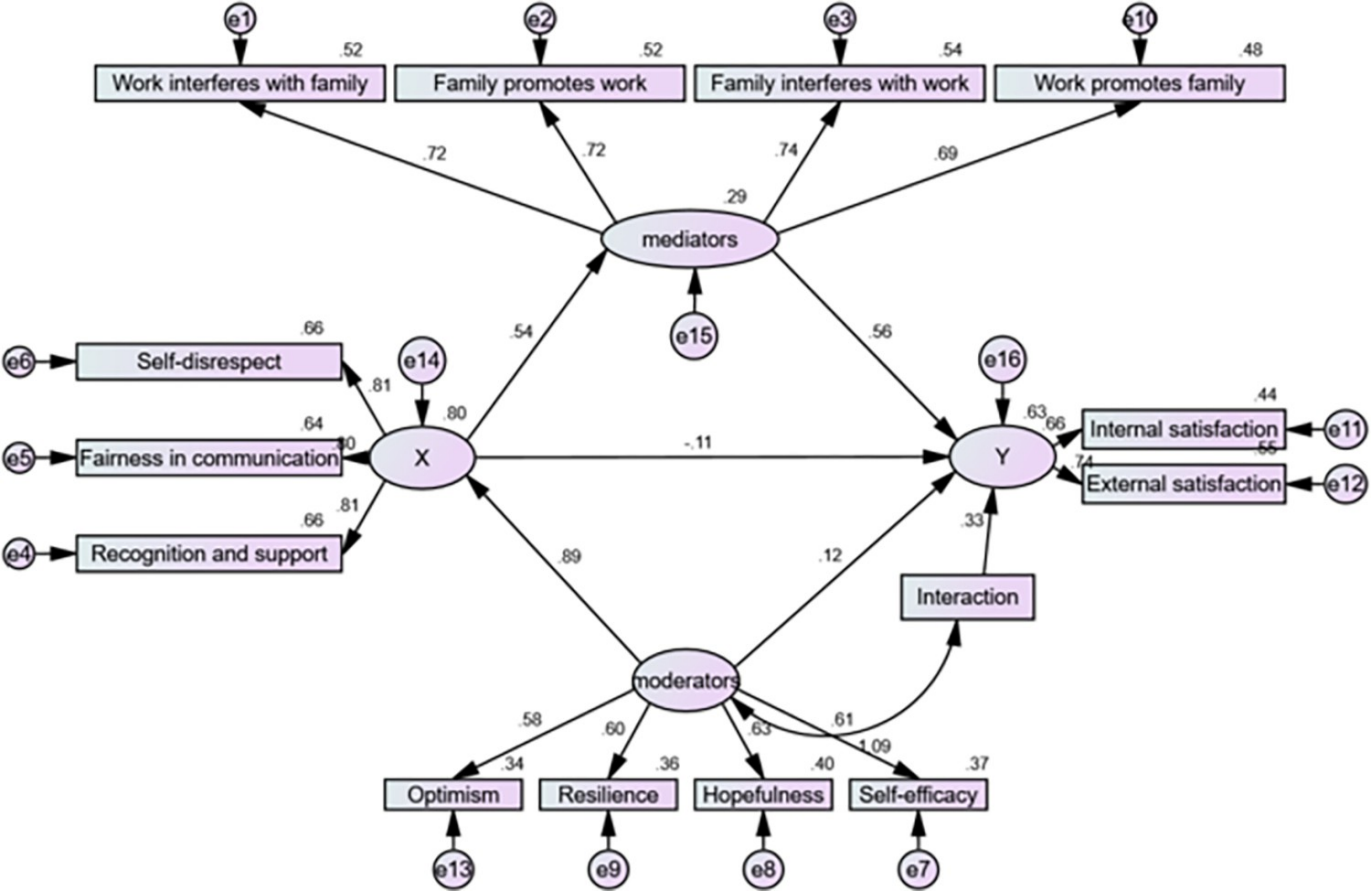
Amos model.

The X mediators and the -Y mediators indicate that the mediating effect was significant, and XY was not significant, indicating that there was complete mediation. The interaction term -Y had a significant effect, representing a significant moderating effect (Fig 1).

## Discussion

The research findings reveal the positive and significant effect of inclusive leadership on job satisfaction. This study also demonstrates the significant mediating and moderating roles of work-family balance and psychological capital. Comparing the current findings with the extant literature shows that numerous studies support the findings in the Chinese context. For example, Nurani et al. (2022) confirmed that an inclusive leadership style has a significant positive impact on entrepreneurs’ work-family balance and psychological empowerment. Work-family balance is a common problem encountered in the process of entrepreneurship, and simply reducing work time or workload is not a good strategy to achieve work-family balance. Sfakianaki et al. (2018) revealed that leadership style has a significant positive correlation with the work attitude, work behavior, and organizational performance of the worker group, which can be generated by encouraging employees to switch their interests to organizational interests. This study adds to the existing literature on the relationship between inclusive leadership style and job satisfaction that is directly related to leadership styles, as well as new evidence regarding the mediating effect of work-family balance and the moderating effect of psychological capital on the relationship between inclusive leadership style and job satisfaction for the group of university workers.

### Theoretical implication

This study has several theoretical implications. First, this study conducts model construction and empirical analysis on the relationship between inclusive leadership and employee satisfaction from the unique perspective of the Chinese cultural context, which enriches the connotation of inclusive leadership research and expands research on indigenous theories of inclusive leadership. At present, competition and organizational changes caused by economic globalization are increasing day by day. Organizations such as universities, which undertake the missions of national production, education, and research, are also facing restructuring. The leaders of universities are also increasingly aware of the importance of the leadership behavior of the leaders themselves in the organizational management of universities. Influenced by traditional Chinese culture, Chinese university leaders often adopt authoritative leadership, paternalistic leadership, and other leadership styles, emphasizing differences between superiors and subordinates and managing with leadership privileges. However, a single leadership method can easily imprison employees’ thinking and inhibit employees’ initiative, which is not conducive to the long-term development of individual employees or even the organization. University leaders can improve their leadership skills by continuously learning a variety of leadership theories, including the inclusive leadership style, and practicing them so that young employees’ job satisfaction can be increased.

Second, this study explores the mechanisms of inclusive leadership on employee satisfaction from the perspective of psychological fields, and the research object is young employees, which further expands and confirms the feasibility of field theory in the field of social psychology (Reed & Lewin, 1951). As China’s educational resources become more and more abundant, with the Internet achieving unprecedented development in China, followed by the input of diverse innovative cultures, such characteristics of the times have made employees born between 1985 and 1995 different from the older generation with respect to their world view, outlook on life, and values. Inclusive leaders tolerate and embrace deviations from traditional practices. The employees born between 1985 and 1995 have distinct personalities, and most of them have received higher education, so they have a wealth of cultural knowledge. Compared with older employees, the knowledge gap between them and their leaders has narrowed. Furthermore, the employees born between 1985 and 1995 prefer an open style of working, with leaders who are willing to listen to their ideas and pay attention to their needs, a working environment that is open and free, and more autonomy at work. Finally, employees born between 1985 and 1995 have strong innovative thinking, the courage to speak up about their unique ideas, hope that the work they are doing is challenging and growth-oriented, and are eager to arouse the leaders’ and their own attention. If an organization wants to manage employees born between 1985 and 1995 well and stimulate their work potential, it needs to effectively change the leadership style according to the personality characteristics of the new generation of employees and use different authoritative management methods to motivate such employees.

Finally, this study contributes by furthering understanding of the mediating role of work-family balance when psychological capital positively moderates the indirect effect of inclusive leadership on improving job satisfaction. Nowadays, people often play multiple roles, and the roles of work and family are closely related. As a matter of fact, individuals who are too invested in their work roles often experience adverse effects on their role in the family. The relationship between the two roles is difficult for employees, so when professionals are facing work-family conflict [36], it is difficult for them to balance work and family. As a source of stress, the work-family conflict will bring a lot of negativity. For example, this kind of pressure will place a burden on the body and mind, resulting in the inability to concentrate on work, negative attitudes, low morale, and then a higher absenteeism rate. In addition, the life order of employees will also be disturbed, and the living standards will be lowered. If not solved well, these problems will continue to intensify, further increasing the pressure on employees and even leading to irrational behavior on their part. Therefore, as work interferes with family life too much, they will experience more negative emotions and a reduction in their sense of job gain at work, especially in terms of physical and material acquisition.

### Practical implication

This study also offers several practical implications for policymakers and university administrators to encourage employee job satisfaction. First, the results of this paper show that inclusive leadership will give young employees a positive emotional experience, enhance their job satisfaction, promote their high level of work engagement, and promote organizational development. This requires university leaders to focus on improving their inclusive leadership style, pay attention to emotional investment in university employees, understand and support the work of university employees, and respect the reasonable needs of university employees. At the same time, employees have a positive attitude toward their job responsibilities because of their job satisfaction, and the definition of job satisfaction is different for everyone [37]. It is necessary to actively clarify the meaning of work to employees, paint a bright future for employees, care about their work and living conditions, establish close contact with employees, realize friendly interaction with employees, and promote employees to form a sense of organizational identity and organizational belonging. This will achieve the purpose of promoting young employees to work wholeheartedly and contribute to the organization. Leaders of universities can rely on and exert their charisma and influence to command, lead, guide, and encourage university employees born from 1985 to 1995 to achieve their work goals.

Second, it can be seen that in addition to understanding employees’ business performance and skill level, universities and leaders should pay more attention to the emotional state of employees. The results show that a high level of psychological capital can enhance the endogenous motivation of employees, thereby promoting the benign and efficient development of the organization. In addition, a low level of psychological capital is not conducive to the generation of employee organizational trust and can easily cause employees to be in a state of depression, slack at work, or even resign, which is harmful to the organization. As a result, universities should pay attention to the emotional experience of young employees. Psychological safety is an internal resource, and inclusive leadership is an external resource that stimulates the development of positive emotions through an open environment [38]. On the one hand, in the process of communicating with employees, leaders should reduce negative feedback to employees, positively support, encourage, and care for employees more, and establish a close reciprocal relationship with them. On the other hand, the organization must unblock communication channels, establish an effective communication mechanism with employees, understand the emotional state of employees, promptly provide a platform for employees to talk, resolve their negative emotions, and avoid the weakening of young employees’ psychological capital by negative emotions.

Finally, universities have a significant impact on an employee’s work-family balance through a variety of factors, so finding out how to promote improved employee performance and prevent work-family conflict is important. It is worth mentioning that, in the context of Chinese culture, repaying parents and ensuring family happiness is the goal and source of motivation for young employees in universities under the age of 40. The findings also confirm that work-family balance plays a mediating role in the relationship between inclusive leadership style and job satisfaction among young university employees. Therefore, to improve the job satisfaction of their young employees, universities need to provide support to meet their young employees’ needs in the family field and promote their work-family balance. For example, providing consulting services and training counseling to effectively deal with work-family relationships to achieve resource compensation, helping young university employees to acquire knowledge, experience, skills, and positive experiences in the family field, and achieving resource overflow, thereby improving job satisfaction and achieving “humanistic care” and “people-oriented management”.

## Conclusion

Based on the research method of empirical analysis, this study focuses on the relationship between inclusive leadership and job satisfaction among young employees by using work-family balance and psychological capital as mediating and moderating variables. The main conclusions of the research are as follows: The more inclusive leadership styles an organization’s leaders have, the more job satisfaction among young workers increases. When organizational leaders have an inclusive leadership style, the level of work-family balance among employees will be increasingly improved. The mediating role of work-family balance between inclusive leadership and job satisfaction is stronger when employees’ psychological capital is at a higher level.

This study implemented a single rating method for data collection from key informants. There may nevertheless be a degree of bias from the employees’ perspective. Future studies may expand the investigation by using the leader’s perspective. Further, implementing a quantitative approach to evaluate the constructs identified represents one of the study’s limitations. The interview approach or a mixed-method approach could provide a more in-depth analysis. Finally, future studies should be conducted in diverse contexts, and different types of organizations should be targeted in order to broaden the domain and increase generalizability.

## Supporting information

S1 Data (XLSX)
